# Comprehensive Raman Fingerprinting and Machine Learning-Based Classification of 14 Pesticides Using a 785 nm Custom Raman Instrument

**DOI:** 10.3390/bios15030168

**Published:** 2025-03-05

**Authors:** Meral Yüce, Nazlı Öncer, Ceren Duru Çınar, Beyza Nur Günaydın, Zeynep İdil Akçora, Hasan Kurt

**Affiliations:** 1 SUNUM Nanotechnology Research and Application Centre, Sabanci University, Istanbul 34956, Türkiye; nazli.oncer@sabanciuniv.edu (N.Ö.); beyzagunaydin@sabanciuniv.edu (B.N.G.); 2 Department of Bioengineering, Royal School of Mines, Imperial College London, London SW7 2AZ, UK; 3 Department of Computer Science & Engineering, Sabanci University, Istanbul 34956, Türkiye; ceren.cinar@sabanciuniv.edu; 4 Department of Materials Science and Nanoengineering, Sabanci University, Istanbul 34956, Türkiye; 5 Department of Molecular Biology, Genetics and Bioengineering, Sabanci University, Istanbul 34956, Türkiye; idil.akcora@sabanciuniv.edu

**Keywords:** pesticide detection, food monitoring, machine learning, 785 nm Raman, 532 nm Raman

## Abstract

Raman spectroscopy enables fast, label-free, qualitative, and quantitative observation of the physical and chemical properties of various substances. Here, we present a 785 nm custom-built Raman spectroscopy instrument designed for sensing applications in the 400–1700 cm^−1^ spectral range. We demonstrate the performance of the instrument by fingerprinting 14 pesticide reference samples with over twenty technical repeats per sample. We present molecular Raman fingerprints of the pesticides comprehensively and distinguish similarities and differences among them using multivariate analysis and machine learning techniques. The same pesticides were additionally investigated using a commercial 532 nm Raman instrument to see the potential variations in peak shifts and intensities. We developed a unique Raman fingerprint library for 14 reference pesticides, which is comprehensively documented in this study for the first time. The comparison shows the importance of selecting an appropriate excitation wavelength based on the target analyte. While 532 nm may be advantageous for certain compounds due to resonance enhancement, 785 nm is generally more effective for reducing fluorescence and achieving clearer Raman spectra. By employing machine learning techniques like the Random Forest Classifier, the study automates the classification of 14 different pesticides, streamlining data interpretation for non-experts. Applying such combined techniques to a wider range of agricultural chemicals, clinical biomarkers, or pollutants could provide an impetus to develop monitoring technologies in food safety, diagnostics, and cross-industry quality control applications.

## 1. Introduction

Pesticides, such as insecticides, herbicides, and fungicides, play an important role in modern agricultural practices by protecting crops from pests and diseases. However, widespread and excessive use of pesticides causes environmental pollution, bioaccumulation, and adverse human health effects [1]. Analytical methods for detecting pesticide residues, such as Gas Chromatography-Mass Spectrometry (GC-MS) [2] and Liquid Chromatography-Mass Spectrometry (LC-MS) [3,4], are known as the gold standard. Various other detection methods, such as immunoassays [5,6], electrochemical detection strategies [7], and optical sensors [8,9], have also been reported in the literature for pesticide detection. Despite their accuracy, these methods require significant technical expertise and laboratory setups, paving the way for developing faster and more user-friendly detection methods.

Raman spectroscopy (RS) has emerged as a promising alternative in sensor studies due to its rapid, non-destructive nature and minimal sample preparation requirements. The Raman effect was observed in 1928 by C. V. Raman and K. S. Krishnan, and also, independently, by G. Landsberg and L. Mandelstam, but the Nobel Prize in Physics for this discovery was won by C.V. Raman [10]. Raman describes the inelastic scattering of photons that provide information about the vibrational, rotational, and other low-frequency modes of molecules, constituting a unique fingerprint for each chemical compound correlated with the presence and number of molecules in a sample. The utility of Raman spectroscopy extends beyond pesticide detection [11] and includes applications in other environmental monitoring applications, clinical diagnostic studies, and quality control processes across various industries [12,13,14]. Compared to traditional detection methods, Raman spectroscopy offers a path to in situ detection, which is particularly useful in agricultural and food safety monitoring [6,8,15].

Recent advances in machine learning (ML) and multivariate statistical analysis methods, such as Random Forest (RF), Support Vector Machines (SVM), and Principal Component Analysis (PCA), have further enhanced the potential of Raman spectroscopy for data classification and residue identification [16]. For example, Sahin et al. (2022) used ML-assisted Surface Enhanced Raman Spectroscopy (SERS) on flexible substrates for rapid and on-site pesticide detection [17]. Similarly, Zhou et al. (2023) proposed an ML-enhanced nanophotonic sensor for ultrasensitive pesticide detection [18]. Guo et al. (2021) applied chemometric analysis and ML in processing large Raman datasets [19], and Chen and Baek (2023) used a hybrid ResNet model to classify Raman spectra from pesticide samples [20]. These methods have the potential to facilitate the automatic interpretation of large and complex spectral datasets, enabling the identification of subtle differences in Raman fingerprints with little expert knowledge. Despite progress, there are gaps in the literature. Current studies have focused on detecting a limited number of pesticide residues, and the data obtained are either inconsistent with other sources in the literature or vary from one measurement/user to another, making the data less adaptable for specific applications. Generating comprehensive, reliable, reproducible Raman fingerprint libraries on standard surfaces, i.e., silicon wafers, for chemicals is essential to ensure accurate classification and quantification.

According to the fresh fruit and vegetable sector report presented by Turkey’s Ministry of Economy in 2017 [21], the most popular fresh fruit and vegetable products in the world include cucumbers, apricots, cherries, peppers, tomatoes, onions, cabbage, gherkins, pepper, zucchini, lettuce, and chicory; Turkey ranked fourth in the world, with a 2.2% share in the production of these products. According to the same report, the countries Turkey exports fresh fruit and vegetables to are the Russian Federation (39%), Iraq (20.3%), Germany (3.4%), and Ukraine (5%). For pesticide selection, Turkey’s most exported fresh fruit and vegetable data were partially investigated, and pesticides frequently used to grow green pepper or cucumber or detected in their production were evaluated. Some pesticides on the list of banned (unlicensed) pesticides but still in use were also taken into consideration.

**Figure 1:**
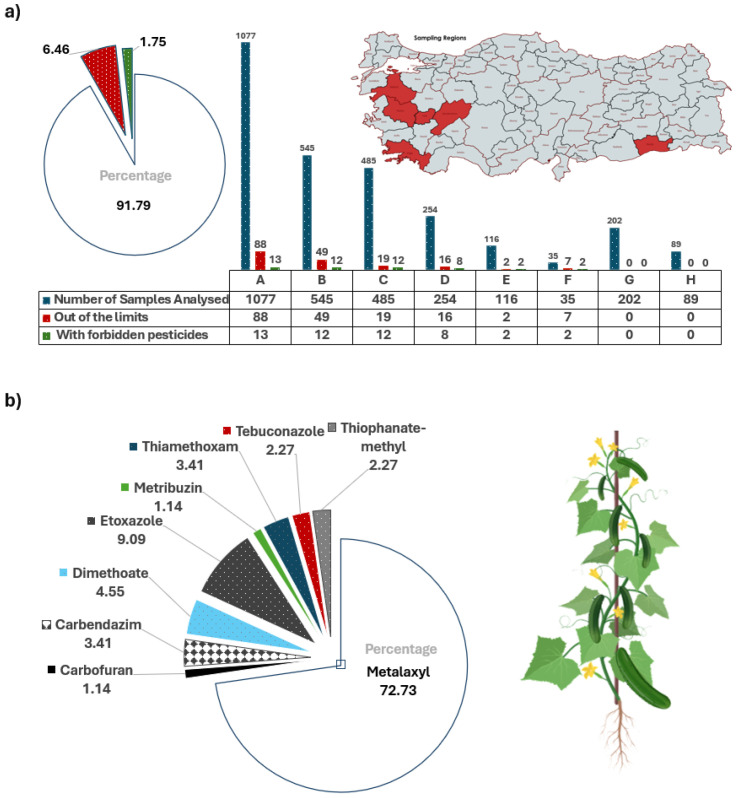
(**a**) Pesticide analysis of cucumber crop samples collected from eight different regions (A–H) of Turkey in 2020 is shown. These data were compiled from the annual (2019–2020–2021) analysis reports of a local agriculture company. (**b**) Percentage distributions of the pesticides in gherkin-type cucumber samples collected from Region A. The sampling map was constituted using mapchart.net.

In Figure 1a, we present the pesticide analysis summary of cucumber crop samples collected from eight different regions (A–H) of Turkey in 2020. These data were compiled from the annual (2019–2020–2021) analysis reports of a local company, which is also one of the major vegetable exporters. The overall dataset presents the analysis of a total of 2803 gherkin-type cucumber samples taken from eight different production regions of Turkey. As can be seen, pesticide rates were found to be above the limits in six of the eight regions (75%) where samples were taken and prohibited pesticide residues were detected in six of them (75%). While 6.46% of all analyzed products failed the pesticide limit test, 1.75% were found to have completely prohibited and harmful pesticide residues. In the 2021 quality analysis report data, which was carried out by increasing the number of regions from 8 to 16, a significant increase was observed in these percentages; in summary, 10.71% of all analyzed products (2914 pieces) failed the pesticide limit test, and 2.37% were found to have completely prohibited and harmful pesticide residues.

In region A, where the largest number of samples (1077) were collected, the distribution of pesticide samples (88 samples) for 2020 is presented in Figure 1b. As can be seen, Metalaxyl ranks first with 72.73%. In the 2021 analysis results of the same region, an increase in the number of pesticide samples was observed with the increasing number of samples. When the pesticide distribution in all regions (from A to H) is examined, Metalaxyl, Chlorpyrifos, Thiamethoxam, Carbendazim, and Etoxazole stand out as the most detected active substances above the limit, in line with the distribution in region A. In the 2021 quality analysis reports made on different types of pepper samples, 36.30% of the 1482 samples examined had above-limit pesticide residues and also heavy metals (lead and cadmium). Among the pesticides, Acetamiprid, Boscalid, and Imidacloprid Metalaxyl/Metalaxyl-M residues, which were also seen in cucumber, were noteworthy. In light of the given datasets, we decided to investigate the following 14 pesticides: Metalaxyl, Chlorpyrifos, Thiamethoxam, Acetamiprid, Cypermethrin, Ametoctradin, Imidacloprid, Etoxazole, Dimethomorph, Chlormequat chloride, Pyrimethanil, Famoxadone, Tebuconazole, and Boscalid.

In this study, we aimed to create a unique Raman fingerprint library of 14 reference pesticide samples using a 785 nm Raman spectroscopy system developed in our laboratory in the 400–1700 cm^−1^ spectral range. The study also compares the Raman spectra of pesticides obtained using a commercial 532 nm Raman instrument to provide a comprehensive characterization dataset to the community. By combining machine learning techniques such as the RF classifier and other multivariate analysis tools such as PCA and Hierarchical Cluster Analysis (HCA), we aimed to produce a reliable Raman spectrum library for the selected pesticides, automate the classification of the samples, and highlight the unique spectral features that distinguish them.

## 2. Materials and Methods

### 2.1. Chemicals

The pesticides listed in Table 1 were purchased from Sigma-Aldrich (St. Louis, MO, USA), and the silicon wafers were obtained from MicroChemicals GmbH (Ulm, Germany).

### 2.2. Instrumentation and Software

The measurements were performed with an in-house Raman spectrometer. The Raman excitation was achieved using a narrow linewidth fiber-coupled laser with a built-in optical isolator, a wavelength of 785 nm, and a maximum power of 400 mW (Cobolt 08-NLD, Hubner Photonics, Solna, Sweden). The Raman excitation and signal collection were performed using a 60× microscopy objective (CFI Plan Fluor 60× 0.85 NA, Tokyo, Nikon, Japan). The Raman signal was later coupled into a multimode optical fiber and delivered to the spectrometer. We have utilized an aberration-free spectrograph (Isoplane SCT320, Princeton Instruments, Trenton, NJ, USA) coupled with a back-illuminated CCD spectroscopy camera (iVaC 316 LDC-DD, Andor, Belfast, UK) for the spectral detection of the Raman signal. The spectral calibration of the spectrograph was maintained using a Ne/Ar lamp (IntelliCal, Princetion Instruments, USA). The wide-field imaging of the sample was performed using a fiber-coupled broadband LED (MBB1L3, Thorlabs, Germany) and color CMOS camera (acA3088-16gc, Basler, Ahrensburg, Germany). The laser power was controlled and monitored using Cobolt Monitor^TM^ software (Hubner Photonics, Solna, Sweden). The raw Raman signal data were collected using Solis spectroscopy software (v.4.32. 30065.0, Andor, Belfast, UK). The spectral resolution of the in-house Raman spectrometer was <1 cm^−1^.

For the in-house Raman spectrometer, we used a laser power of 20 mW (coupled to the fiber) and collected the signal with an integration time of 30 s.

For commercial instruments (inVia™, Wotton-under-Edge, Renishaw, UK), the measurements were performed in the 200–2200 cm^−1^ spectral range using a 50× microscope objective at a wavelength of 532 nm and laser power of 7.5–15 mW with an integration time of 20 s. The spectral resolution of the commercial Raman spectrometer was <1 cm^−1^.

The Raman spectra of the selected pesticides were collected in powder form in both commercial and in-house Raman spectrometers. At least 23 measurements were performed for each pesticide to ensure data accuracy and quality. Multiple spectra were collected for each pesticide, and the average of each spectrum was presented in the graphs to minimize the variability due to sample heterogeneity and other technical reasons. The averaged spectra were normalized for the final representation.

### 2.3. Data Processing

The analysis of Raman spectra consisted of multiple steps. The first step is to perform data pre-processing to remove distortions in the spectra and make peaks more prominent. Python’s RamanSPy library (v0.2.10, https://ramanspy.readthedocs.io/en/latest/, accessed on 1 September 2024) is used for pre-processing purposes [22]. This library provides pipelines, a set of pre-processing methods that form a reproducible and automated pre-processing tool [23]. These methods were tested on the spectra of Metalaxyl (25 spectra from various regions). Kindly refer to Supplementary Information, Figure S1.

IARPLS (Improved Asymmetrically Reweighted Penalized Least Squares) was chosen for the baseline correction as it preserves the peaks and smooths the non-peaked areas. The pipeline methods used are the Whittaker Heyes despiking, IARPLS baseline correction, and the Savitsky–Golay denoising method. The dataset was normalized using the Min-Max (0–1) method using the same pipeline to avoid difficulties due to the magnitude of the intensity values. All pesticide spectra were pre-processed using the pipeline in Python. After all these pre-processing steps, the Random Forest Classifier algorithm, a machine learning tool, was used to observe and measure the prominence of the Raman peaks and classify the model.

Spectral data were averaged and illustrated using OriginPro 2024 software (spectra collected from 25 regions for Thiamethoxam and Dimethomorph and 23 areas for other pesticides). Multivariate statistical techniques such as PCA and HCA were calculated on OriginPro 2024 to distinguish between 14 pesticide samples. PCA with full cross-validation in the spectral range of 1700–400 cm^−1^ was performed on the data pre-processed through Python using the pipeline. HCA was then performed using Ward’s algorithm.

The baseline corrected, normalized, and averaged data (n ≥ 20) for all pesticides from both instruments (785 nm and 532 nm Raman Instruments) were provided publicly on the website given below. The algorithm used for the machine learning study was also publicly available on the same website link: https://github.com/meralyucekurt/Raman-Fingerprints-of-Pesticides (accessed on 25 February 2025).

### 2.4. Testing on Spiked Agricultural Produce

The fresh cucumber, green pepper, and wheat flour were obtained from a local supermarket. We have utilized the QuEChERS (Quick, Easy, Cheap, Effective, Rugged, and Safe) method which is widely used for pesticide analysis [24] to prepare the pesticide analysis samples. A total of 10 g of each food sample is homogenized and 10 mL of acetonitrile is added along with the spike pesticides at a concentration of 10^−5^ M in a 50 mL centrifuge tube. The samples were shaken vigorously using a vortex generator for 2 min each. Later, 4 g of anhydrous magnesium sulfate, 1 g of sodium chloride, and 1 g of trisodium citrate were added to the samples. The samples were agitated vigorously again and then centrifuged at 3000 rpm for 5 min. The raw extract was later filtered using 0.45 µm syringe filters. A total of 5 µL of each filtered extract sample was applied on a clean Si wafer and quickly dried on the substrate. The samples were later illuminated by the in-house Raman spectrometer at a power of 200 mW. The Raman signal was collected for a duration of 120 s.

## 3. Results and Discussion

### 3.1. Instrument Design and Characteristics

**Figure 2:**
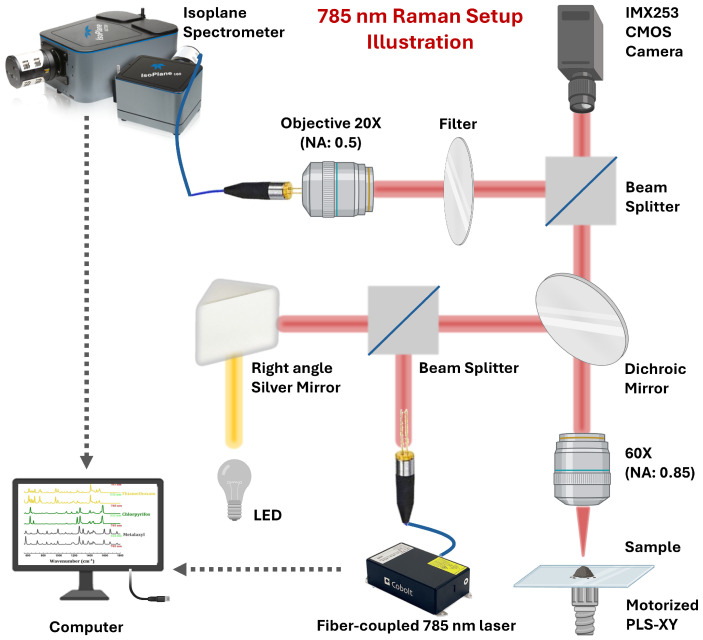
Schematic diagram of the developed house Raman instrument showing the 785 nm wavelength monochromatic laser, the system (fiber) that carries the light from the laser source to other parts of the system, and a turning mirror that guides the optical path and ensures the laser is transmitted accurately. It also illustrates the light source of the system LED, a dichroic mirror, which directs the laser light at the sample and recovers the light from Raman scattering and transmits it to the detection system; a beam splitter, which separates or combines the light paths between the laser and the detected Raman scattering; a filter, which blocks unwanted wavelengths during Raman measurements and transmits only the target wavelengths; a camera (IMX253 CMOS) connected to the Wi-Fi network used for imaging and monitoring of the sample, ×60 objective to collect the Raman scattering from the sample and transfer it to the optical system; a sample scaffold (stage) placed on a mobile platform (PLS-XY) controlled by a joystick to facilitate precise positioning of the sample, ×20 objective that focuses the laser light to deliver high intensity energy to the sample; a CCD detector (iVac 316 LDC-DD) that detects the Raman scattering obtained from the sample, and detects and measures the light; and a computer (PC) that analyzes, displays, and records the spectral properties of the light detected with this system.

In this study, we aimed to create a library by profiling the Raman fingerprints of 14 pesticide samples using a 785 nm Raman spectroscopy system developed in our laboratory in the 400–1700 cm^−1^ spectral range. The system consists of a high-resolution spectrometer, a CCD detector, and a 785 nm laser source, which is known to reduce fluorescence interference compared to shorter wavelengths such as 532 nm. The optical schematic of the in-house developed Raman spectrometer is presented in Figure 2.

In the system, a spectrally pure narrow linewidth diode with a wavelength of 785 nm and a maximum power of 400 mW was used as a laser source (Cobolt 08-NLD, Hubner Photonics, Sweden). This laser was chosen for its wide wavelength range, low noise, and high performance. The laser beam is carried by the FC/PC (Ferrule Connector/Physical Contact) fiber and splits in the prism (BSW29R, Thorlabs, Germany); part of the split beam goes to the turning mirror (CCM1-P01/M, Thorlabs, Germany).

The turning mirror is positioned so that the LED source light (PI, Intellical) and the laser light pass through the same optical path. The other part of the light comes through a filter to a dichroic mirror (Thorlabs, AC254-150-AB, f = 150 mm). The dichroic mirror directs the laser light onto the sample and transmits it to the optical lens (Nikon Plan Fluor λ 60×, 20.0 mm WD, 0.20 NA) so that the scattered Raman light is collected back. The scattered Raman light is transmitted to the detection system through an ×20 (Nikon Plan Fluor 0.30 mm WD, 0.85 NA) lens. As a result, a Raman spectrum in the range 400–1700 cm^−1^ is collected by a CCD sensor (Andor iVac 316 LDC-DD) and an imaging spectrometer (PI, Isoplane SCT320) integrated with this sensor.

Furthermore, the light path through the dichroic mirror is connected to a CMOS camera (Basler, acA4112-30um, 12.3 MP, IMX253, Basler, Ahrensburg, Germany) with Wi-Fi networking capability, which is used for visualization and monitoring of the system. The sample is moved by a 2D joystick in the XY plane (Thorlabs, PLS-XY) and a 1D joystick in the z-direction (Thorlabs ZFM2030).

### 3.2. Raman Fingerprint Spectra of the Pesticides

**Figure 3:**
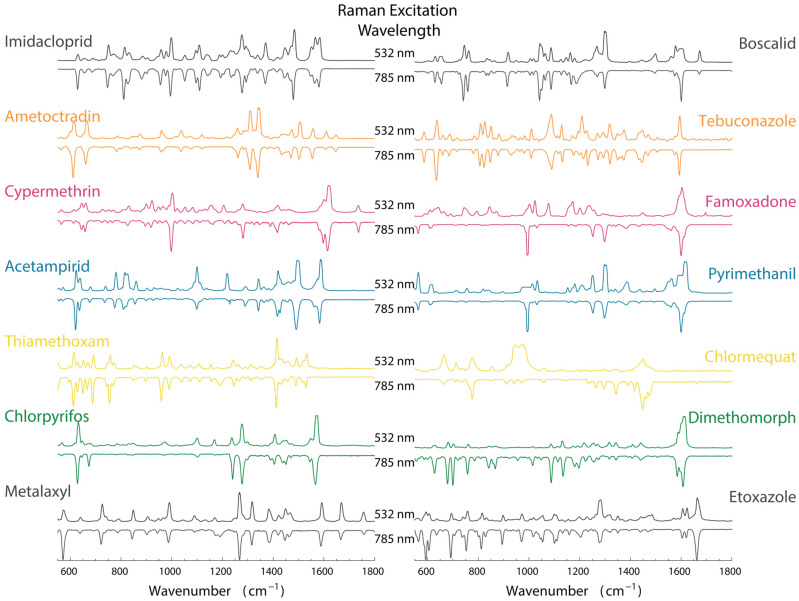
Pairwise mirror spectra of 14 pesticide reference samples. The measurements were performed with a custom-developed 785 nm Raman spectrometer and a commercial 532 nm Raman instrument. The pesticides were measured directly from powders on pre-cleaned, disposable silicon wafers. The spectra are presented in the spectral range of 550–1800 cm^−1^. Means and standard deviations of the spectra were collected from 23 regions for Dimethomorph and 25 areas for other pesticides.

**Figure 4:**
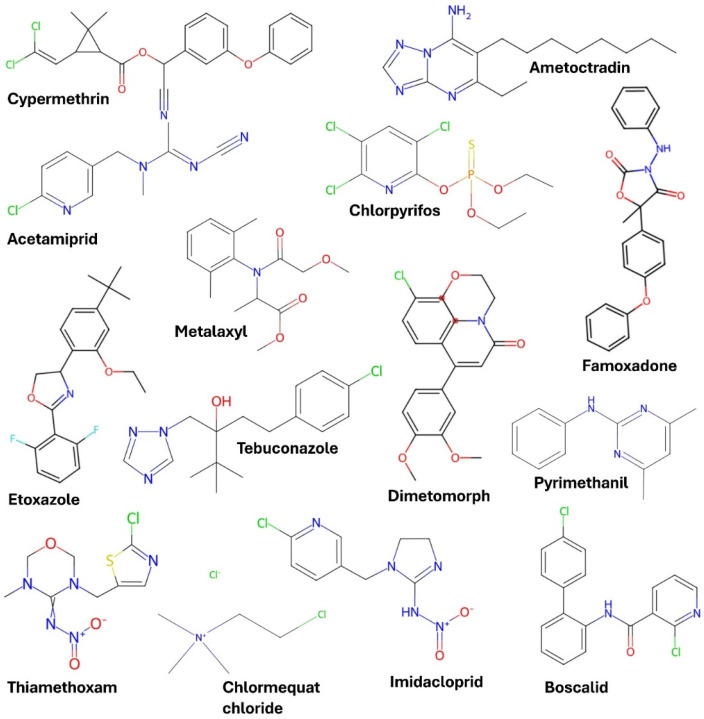
Chemical structures of the pesticides. High-resolution images were extracted individually from the Chem Spider website [27].

Raman spectra data were acquired using the in-house developed Raman instrument and a benchtop Raman (inVia™ confocal) to characterize and detect the fingerprint spectra and fundamental vibrational bands of the standard pesticide powders. The result of Raman analyses is presented in Figure 3. Comparative use of these two instruments aimed to validate the in-house system’s performance and provide a comprehensive spectral library for pesticide classification purposes. Paired instrument comparisons have been used previously in Raman spectroscopy studies to show the influence of excitation wavelength on spectral quality and signal intensity [25,26]. Both systems were used to measure the Raman spectra of 14 pesticide powders. The chemical structures of the pesticides were extracted individually from the Chem Spider website [27] and represented in Figure 4.

The data obtained from both Instruments clearly showed the characteristic bands of pesticide powders (Figure 3). The results obtained were majorly consistent, with some differences in peak intensities and Raman shifts among spectrums between the two instruments (571 to 569 cm^−1^; 628 to 621 cm^−1^; 959 to 966 cm^−1^; 621 to 626 cm^−1^; 998 to 1001 cm^−1^; 660 to 641 cm^−1^; 1278 to 1271 cm^−1^; 1278 to 1291 cm^−1^; 1607 to 1609 cm^−1^; 775 to 775 cm^−1^; 1298 to 1297 cm^−1^; 1600 to 1603 cm^−1^; 634 to 630 cm^−1^; and 1600 to 1594 cm^−1^ for Metalaxyl, Chlorpyrifos, Thiamethoxam, Acetamiprid, Cypermethrin, Ametoctradin, Imidacloprid, Etoxazole, Dimethomorph, Chlormequat chloride, Pyrimethanil, Famoxadone, Tebuconazole, and Boscalid, respectively). Slight variations are expected due to differences in wavelength, reduced applied power, laser sources, and unintentional user errors.

The relative intensity of certain vibrational peaks was also affected by the excitation wavelength. For example, Imidacloprid showed stronger intensity for the 1105 cm^−1^ peak under 785 nm excitation compared to 532 nm excitation. On the other hand, Cypermethrin-related peaks at 1001 cm^−1^ and 660 cm^−1^ were more pronounced under 532 nm excitation, which was attributed to potential resonance effects at shorter wavelengths. Such variations seem to be consistent with the findings from previous Raman studies that shorter wavelengths, such as 532 nm, can induce resonance enhancement in π-conjugated molecular systems, possibly interacting more with the ground state vibrational modes [25,27,28]. These differences also indicate the importance of wavelength selection when analyzing pesticide residues using Raman spectroscopy. Additionally, when real food samples are used, there is a greater likelihood of background noise or background fluorescent signals. Fluorescence often obscures Raman peaks and raises the baseline, especially when shorter excitation wavelengths such as 532 nm are used. In the present study, the 785 nm system produced clearer spectra with lower background fluorescence, especially in samples such as Chlorpyrifos and Dimethomorph. Similar trends have been reported in studies using near-infrared (NIR) excitation, where reduced fluorescence allows more sensitive measurement of Raman shifts in complex organic samples [24,27]. These comparative analyses established the performance and reliability of the in-house Raman system, setting a precedent for developing lower-cost, portable Raman instruments for agricultural and environmental applications. The 532 nm system showed higher sensitivity for certain peaks, while the 785 nm system provided slightly superior background suppression, making it ideal for fluorescence-prone samples. Data from the comparative analysis provided a clear database and guidance on the selection of appropriate excitation wavelengths for future Raman-based pesticide sensor systems.

### 3.3. Peak Positions and Vibrational Assignments of the Pesticides

Finally, Table 2 presents the major and minor Raman peaks of the pesticides and the corresponding peak assignments. Each pesticide is associated with specific peak positions, vibrational assignments, and references for the data. The results showed that the house Raman instrument successfully measured Raman ‘fingerprint’ spectra for pesticide powders and was compatible with a benchtop Raman instrument. As a note, peak assignments for Metalaxyl, Ametoctradin, and Famoxadone were reported for the first time in this study.

Raman spectral analysis of 14 pesticides provides a comprehensive framework to identify and characterize these compounds based on their molecular vibrations. The results highlight distinct Raman peaks associated with their unique molecular structures and functional groups for each pesticide, which can be used for effective detection and differentiation. Each pesticide showed characteristic Raman peaks corresponding to specific vibrational modes, including bond stretching, bending, and ring deformations. For example, Metalaxyl exhibited peaks at 572, 726, and 990 cm^−1^, which were attributed to skeletal ring deformations and symmetric trisubstituted ring stretching, while higher frequency modes at 1591 and 1668 cm^−1^ were attributed to aromatic C=C stretching. Similarly, Chlorpyrifos showed notable peaks at 628 cm^−1^ (P=S stretching) and 1567 cm^−1^ (C=C ring stretching), highlighting the importance of phosphorus and aromatic groups in its spectral profile. The analysis revealed that key functional groups play an important role in the spectral properties of these pesticides.

For example, the presence of halogens like chlorine was evident in Acetamiprid (621 cm^−1^, C-Cl stretching) and Chlormequat (775 cm^−1^, C-Cl vibration). Similarly, Cypermethrin and Imidacloprid exhibited peaks related to C=N and C-N stretching, indicating nitrogen-containing groups that are critical for their classification as insecticides. Peaks associated with sulfur-containing groups, such as C=S stretching in Etoxazole (692 cm^−1^), also highlighted their importance in distinguishing pesticides with similar structural backbones.

Aromatic and ring-related vibrations were prominent in the Raman spectra, highlighting their usefulness in characterizing pesticides. Many pesticides, including Tebuconazole and Famoxadone, exhibited significant peaks in the range of 1000–1600 cm^−1^ associated with phenyl and other aromatic ring extensions. For example, Tebuconazole showed peaks at 634 and 1593 cm^−1^ corresponding to phenyl moiety vibrations, while Famoxadone showed monosubstituted aromatic ring extension at 1603 cm^−1^. These peaks serve as critical markers for the identification of aromatic-based pesticides. The distinct Raman spectral features of these pesticides highlight the effectiveness of the technique in their detection and classification.

The high specificity of Raman peaks, e.g., P=S stretching in Chlorpyrifos and aromatic C-N stretching in Ametoctradin, facilitates the discrimination of structurally similar pesticides. Moreover, the data provides valuable insights for developing machine learning models to classify pesticide residues based on their Raman spectral fingerprints. For example, unique peaks in Thiamethoxam (611, 650, and 959 cm^−1^) and Dimethomorph (627, 1584, 1607 cm^−1^) can serve as training features for accurate identification in complex mixtures. Despite its high specificity, some challenges remain in distinguishing pesticides with overlapping spectral features, especially those with similar functional groups. For example, multiple pesticides exhibited peaks associated with C=C and C=N stretches, requiring additional spectral pre-processing or advanced chemometric techniques to improve classification accuracy.

### 3.4. Spectral Analysis of Pesticides Using Multivariate Statistical Analyses: Principal Component Analysis (PCA) and Hierarchical Cluster Analysis (HCA)

Multivariate statistical methods were applied to the pesticide data (Raman 785 nm only) that was previously baseline corrected, averaged, and normalized. The main challenge is the size and amount of the spectral data, making perception and interpretation difficult. Techniques such as PCA and HCA remove this bottleneck, reducing the size of the data and making it easier to interpret [65,66,67]. One of these techniques, PCA, was used to capture the similarities and differences of the Raman spectra obtained from 14 pesticides.

**Figure 5:**
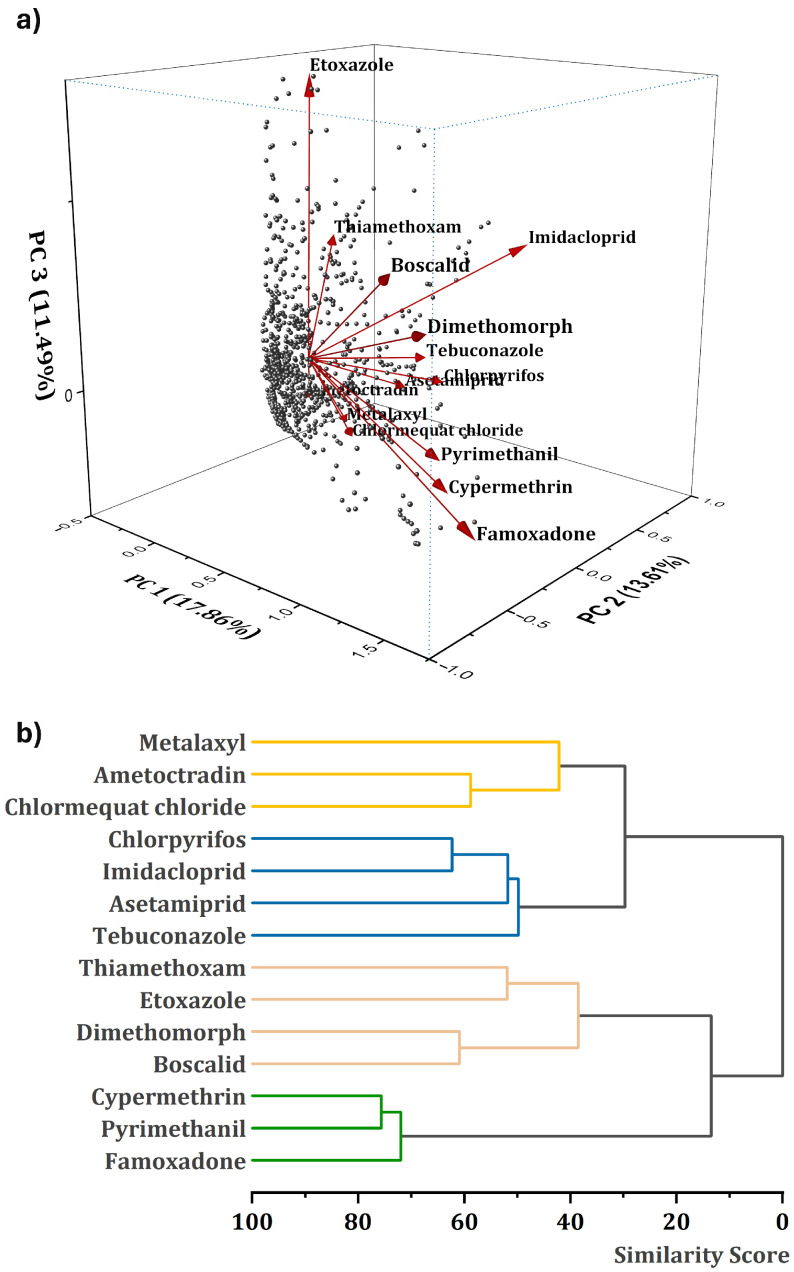
PCA plot (**a**) and HCA dendrogram (**b**) illustrate the multivariate statistical analysis of average Raman spectra from 14 pesticide reference samples. Average Raman spectra data from at least 23 measurements were used for the analyses. The PCA plot displays the distribution of pesticide samples in the reduced dimensional space defined by the first three principal components, highlighting the high variance captured in the spectral data. Each point represents a different pesticide, with proximity indicating similarity in spectral characteristics. The HCA dendrogram provides a hierarchical clustering of the pesticides based on their spectral similarities using Ward’s method to demonstrate how samples group together.

The PCA method was applied to the Raman spectral data of 14 pesticides to reduce dimensionality and identify primary sources of variance. Since a two-component analysis was not found sufficient, a three-dimensional component analysis was performed and demonstrated in Figure 5a. The first three principal components (PC1, PC2, and PC3) explained 42.4% of the combined variance, while their individual contributions were 17.64% (PC1), 13.85% (PC2), and 10.91% (PC3), respectively. Eigenvalues for PC1, PC2, and PC3 were 0.05835, 0.04582, and 0.0361, indicating that the variance was clearly distributed over more than one component.

Analysis of loading plots revealed that Famoxadone, Cypermethrin, Pyrimethanil, and Dimethomorph made strong positive contributions to PC1, indicating that these pesticides were the main drivers of the variance along this axis. For PC2, Imidacloprid, Chlorpyrifos, and Acetamiprid made the most significant contributions, while Boscalid and Etoxazole showed the opposite relationships, exhibiting negative loadings.

The contribution of individual pesticides to PC3 was more distributed, with substantial effects from Etoxazole, Imidacloprid, and Boscalid. The cumulative variance explained by the first three components suggests that multiple hidden factors underlie the variation in Raman spectra, necessitating consideration of additional components or closer examination of pesticide-specific spectral features. Visualization of PC1 and PC2 reveals distinct clustering of pesticides and demonstrates their differentiation based on spectral features.

To further assess the spectral relationships, HCA was performed using the Ward method with a correlation-based distance metric. HCA revealed at least four distinct clusters, as shown in Figure 5b: Cluster 1 contained Metalaxyl, Chlorpyrifos, Acetamiprid, Imidacloprid, and Tebuconazole; Cluster 2 grouped Ametoctradin and Chlormequat chloride; Cluster 3 consisted of Thiamethoxam, Etoxazole, Dimethomorph, and Boscalid; and Cluster 4 contained Cypermethrin, Pyrimethanil, and Famoxadone.

The clustering pattern reflects similarities in spectral features, molecular structures, or vibrational modes and suggests possible similarities in Raman-active functional groups among pesticides within the same cluster. The combined use of PCA and HCA provided complementary insights, with PCA highlighting the most effective pesticides driving the spectral variance. At the same time, HCA revealed natural groupings based on similarity in the multivariate Raman spectral space. These results provided a basis for future classification and identification strategies.

While the joint use of PCA and HCA gives useful information about the underlying structure of Raman spectral data, several limitations should also be noted here. First, the total variance explained by the first three principal components is only 42.4%, indicating that a large portion of the variance is distributed among higher-order components, suggesting that additional principal components may be required to fully capture the variation in the data, complicating visualization and interpretability. Additionally, PCA requires standardization of the data, and any outliers in the dataset may disproportionately affect the results. In the current scenario, datasets include replicates that naturally vary from one measurement to another due to the interaction of the excitation sources with the molecules at each measurement. For HCA, the choice of distance metric (correlation) and linkage method (Ward’s) affects the clustering result, and alternative metrics or methods may produce different groupings. Moreover, HCA does not inherently verify the number of clusters, relying on a slightly subjective interpretation of the dendrogram, introducing a potential bias. Finally, chemical interpretation of PCA loadings and cluster assignments can be challenging because the influence of Raman peaks on components is not always straightforward, especially when multiple pesticides share overlapping vibrational properties, necessitating the inclusion of additional analyses for more reliable classification. To further enhance the classification and prediction ability of pesticide identification, machine learning algorithms were applied to Raman spectral datasets in the next section to build more robust and automatic classification models.

### 3.5. Spectral Analysis of Pesticides Using Machine Learning Techniques: Random Forest Classifier

The dataset of 14 pesticides was classified using the Random Forest Classifier algorithm. The Random Forest Classifier algorithm improves classification accuracy by growing multiple decision trees and selecting the most popular class for classification. As each tree is grown with a randomly selected subset of the training dataset or different combinations of its features, the diversity and robustness of the model are increased. This approach significantly improves classification accuracy and provides a powerful method to accurately classify pesticides based on different spectral features [68].

The application of machine learning models for classification tasks based on Raman spectra is increasing in the literature. However, this number still needs to be higher on pesticide classification, especially using many classes and Random Forest Classifiers. Bellantuono et al. worked with several machine learning models, Random Forest, XGBoost, Support Vector Machine, and Gaussian Naïve Bayes, to classify healthy/benign and carcinoma spectra. They showed that the best algorithm based on the AUC score is the Random Forest Classifier, trained based on spectra peaks and evaluated on 100 runs [13]. Similar to this approach, the peaks were used for the classification task in this paper. As mentioned above and shown by Bellantuono et al., the algorithm outperforms others when the data are high-dimensional. Since this model’s performance is high on complex spectra, the intuition was to adapt it to solve the multi-class classification of 14 pesticides.

The adaptation process included pre-processing of spectra so that the signature peaks are more observable and feature selection, i.e., the three highest peak positions and intensities for each 200 cm^−1^ Raman shift (please refer to Supplementary Information, Figure S2). After obtaining these features, the normalized data are trained and evaluated with Cross-Validation using a Random Forest Classifier object. This method, which improves the reliability of model performance evaluation, randomly splits the data into parts and uses one part for testing and the remaining parts for training. In subsequent iterations, a different part is used for testing, and the accuracy is calculated as an average across all iterations, avoiding overfitting and ensuring more reliable results [69]. For cross-validation, 70% of the total dataset was chosen as the training set, and the remaining 30% was used for testing in each iteration. Thus, switching the test sets performed a total of three-fold cross-validation.

The results are promising since the model can distinguish all 14 pesticides and correctly label all samples in the dataset. Future enhancements to feature selection, such as peak width or thresholding, can also be considered for further applications of the classification task.

**Figure 6:**
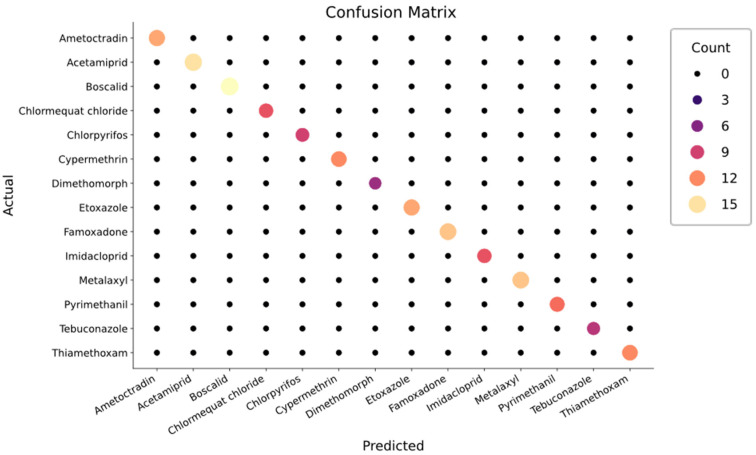
Confusion matrix was obtained by comparing the predicted labels acquired from the trained Random Forest Classifier model with the actual class labels of the pesticide test set. Each cell in the matrix represents the number of samples classified into a specific category (true class) against the predicted categories (predicted class). The diagonal elements indicate correct classifications, while off-diagonal elements represent misclassifications. The overall accuracy, precision, and recall metrics can be derived from this matrix, facilitating an understanding of the model’s effectiveness in distinguishing among different pesticide Raman fingerprint spectra.

The results from the cross-validation are shown using a ‘confusion matrix’ for the classifications in the test set, as depicted in Figure 6. The x-axis represents the predicted classifications, and the y-axis shows the actual labels. Each cell in the matrix indicates the number of samples for the class predicted by the model (x-axis) and the actual class (y-axis). For example, in the matrix at position (0,0), the circle represents “11”, which indicates that the model correctly predicted all eleven samples of Ametoctradin in the Ametoctradin class. Placing all samples along the diagonal of the matrix demonstrates that all samples in the test set were classified correctly. The circle’s size, color, and density represent the number of test samples classified. As the density increases, the color changes slightly from black to yellow. The model’s accuracy, precision, F1 score, and sensitivity are 100%. This high performance is due to the different data used for training and testing, i.e., the Raman peaks [50].

### 3.6. Testing of the Machine Learning Model and Raman Spectroscopy Fingerprint Database in Spiked Pesticide Samples: Metalaxyl and Chlormequat Chloride

To validate the both machine learning model and the in-house Raman spectrometer, we prepared homogenized samples of cucumber, green pepper, and wheat flour, and spiked these samples with a concentration of 10 µM metalaxyl (for cucumber and green pepper) and chlormequat chloride (for wheat flour) pesticides. We used the QuEChERS method to extract the pesticides from these samples. The final food samples later were measured on Si wafer substrates. We have used a higher laser power level and higher integration time for the CCD sensor to detect the significantly lower Raman signal compared to the powder form. The laser power was increased to 200 mW. The CCD array integration time was increased to 120 s.

**Figure 7:**
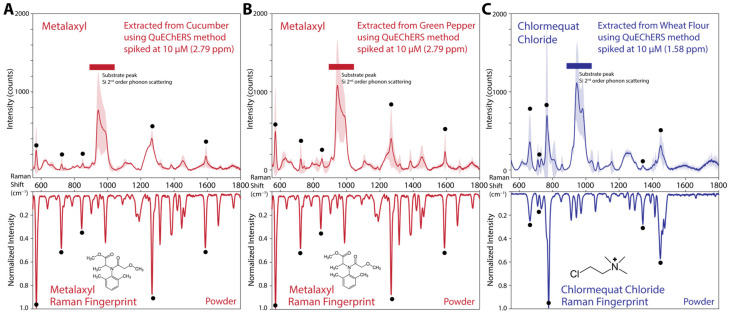
(**A**) The Raman spectrum of 10 µM (2.79 ppm) spiked metalaxyl pesticide extracted from cucumber samples using QuEChERS method. The normalized Raman fingerprint of metalaxyl in powder form was presented at the bottom as inverted spectrum. (**B**) The Raman spectrum of 10 µM (2.79 ppm) spiked metalaxyl pesticide extracted from green pepper samples using QuEChERS method. (**C**) The Raman spectrum of 10 µM (1.58 ppm) spiked chlormequat chloride pesticide extracted from green pepper samples using QuEChERS method. The normalized Raman fingerprint of chlormequat chloride in powder form was presented at the bottom as inverted spectrum. In all spiked sample spectra, the shaded area represents standard deviation (±σ) of seven measurements from independent sites. The black circles designate the five most prominent peaks in both spiked sample and powder reference. All samples were measured in semi-dried form on Si wafer.

The acquired Raman spectra of these spiked samples were processed using the same data processing pipeline. We have observed a notable characteristic Si substrate peaks at 521 cm^−1^ and 900–1000 cm^−1^. However, the spiked sample Raman active resonances were strong enough to detect even at a concentration of 10 µM as shown in Figure 7. The ML model successfully identified both pesticides accurately.

This concentration coincides with a ppm level of 2.79 and 1.58 for metalaxyl and chlormequat chloride, respectively. These values are quite close to the legal residual limits enforced by the EU. The residue limit of metalaxyl in table grapes is 1.5 ppm [70]. The residue limit of chlormequat in rapeseed and wheat is at 7 ppm. However, the legal limits of some pesticides are below 10 ppb in specific agricultural produce. Further development of signal enhancement methodologies such as surface-enhanced or plasmon-enhanced Raman spectroscopy can be implemented in the future to probe sub-ppm level pesticide residues [71].

## 4. Conclusions

The current study introduces a 785 nm custom-built Raman spectroscopy instrument for sensing and characterization applications. This instrument, operating in the 400–1700 cm^−1^ spectral range, is designed to reduce fluorescence interference and improve signal clarity compared to shorter wavelengths like 532 nm. Here, we analyzed and classified 14 different pesticide samples using a 785 nm Raman spectroscopy instrument designed and installed in our laboratory, as well as machine learning techniques. The main findings include a unique Raman fingerprint library that provides detailed molecular information about these pesticides and facilitates their differentiation based on their spectral properties, achieved with multivariate analysis and ML techniques. This library serves as a comprehensive dataset for the identification and classification of pesticides based on their spectral properties.

On the other hand, some important limitations of the study should be considered for future research. For example, focusing on only 14 pesticides limits the scope of the findings; therefore, independent studies covering a wider range of chemicals are needed. Furthermore, although our custom-designed Raman instrument performed well, small differences were observed compared to a commercial 532 nm Raman instrument, indicating potential signal variability due to wavelength and laser power differences. Furthermore, although machine learning significantly helps in data interpretation, algorithms need to be carefully selected and optimized. In the future, we will further validate the effectiveness of the developed Raman spectroscopy and machine learning techniques by applying them to real food samples. We anticipated the undesired fluorescence originating from a laser wavelength of 532 nm and opted for a suitable low-fluorescence laser wavelength of 785 nm. It is also imperative to develop compatible sample preparation routines for improving the detection limit and reproducibility of future applications to real-world samples. These pesticides will be measured in agricultural products, especially in pepper and cucumber, to evaluate their performance in complex matrices and practical conditions. This step is critical to demonstrating the applicability of the Raman fingerprint library in real-life scenarios and will contribute to food safety, environmental protection, and monitoring studies.

The application of Raman spectroscopy and machine learning techniques to a broader range of applications, such as agrochemicals, clinical biomarkers, or contaminants, has the potential to significantly improve detection and monitoring technologies in various fields. For example, in food safety, these methods can increase the reliability and efficiency of safety assessments by enabling rapid and accurate detection of contaminants. In clinical diagnosis, non-invasive and sensitive analysis of biomarkers can enable earlier disease detection and improved patient outcomes. Furthermore, in cross-industry quality control, these techniques can provide detailed information on the structure or composition of materials, ensuring compliance with safety standards and improving product quality. Overall, integrating these advanced analytical tools can provide more robust and real-time monitoring solutions, spurring innovation and improving public health outcomes.

## Figures and Tables

**Table 1:** List and classification of pesticides for which Raman spectra were obtained.

No	Pesticide	Type	Chemical Structure	CAS Number
1	Metalaxyl (*N*-(2,6-Dimethylphenyl)-*N*-(methoxyacetyl)-DL-alanine methyl ester)	Fungicide	Acylalanine	57837-19-1
2	Chlorpyrifos (diethyl-d10)	Insecticide	Organophosphate	2921-88-2
3	Thiamethoxam (3-(2-Chloro-5-thiazolylmethyl)tetrahydro-5-methyl-*N*-nitro-4*H*-1,3,5-oxadiazin-4-imin)	Insecticide	Neonicotinoid	153719-23-4
4	Acetamiprid (N-(6-Chloro-3-pyridyl methyl)-N-cyano-N-methyl acetamidine)	Insecticide	Neonicotinoid	190604-92-3
5	Cypermethrin (5-Ethyl-6-octyl[1,2,4]triazolo[1,5-*a*]pyrimidin-7-amine)	Insecticide	Pyrethroid	52315-07-8
6	Ametoctradin (5-Ethyl-6-octyl[1,2,4]triazolo[1,5-*a*]pyrimidin-7-amine)	Fungicide	Triazolopyrimidine	865318-97-4
7	Imidacloprid	Insecticide	Neonicotinoid	138261-41-3
8	Etoxazole ((*RS*)-5-*tert*-Butyl-2-[2-(2,6-difluorophenyl)-4,5-dihydro-1,3-oxazol-4-yl]phenetole 4-(4-*tert*-Butyl-2-ethoxyphenyl)-2-(2,6-difluorophenyl)-4,5-dihydrooxazole),	Acaricide	Diphenyl oxazoline	153233-91-1
9	Dimethomorph	Fungicide	Morpholine	110488-70-5
10	Chlormequat chloride ((2-Chloroethyl)trimethylammonium chloride),	Plant Growth Regulator	Quaternary ammonium compound	999-81-5
11	Pyrimethanil (2-Anilino-4,6-dimethylpyrimidine)	Fungicide	Anilinopyrimidine	53112-28-0
12	Famoxadone (3-Anilino-5-methyl-5-(4-phenoxy phenyl)oxazolidine-2,4-dione)	Fungicide	Oxazolidinedione	131807-57-3
13	Tebuconazole (1-(4-Chlorophenyl)-4,4-dimethyl-3-(1*H*-1,2,4-triazol-1-ylmethyl)-3-pentanol)	Fungicide	Triazole	107534-96-3
14	Boscalid (2-Chloro-*N*-(4′-chlorobiphenyl-2-yl)-nicotinamide)	Fungicide	Nicotinamide	188425-85-6

**Table 2:** Peak assignment of pesticides.

Pesticide	Raman Peak Positions (cm^−1^)	Vibrational Assignments	Additional Minor Peaks (cm^−1^)	Vibrational Assignments	References
Metalaxyl	572, 726, 990, 1266, 1317, 1591, 1668	Skeletal ring deformations (571, 726), symmetric tri-substituted ring stretch υ(C=C) (990), in-plane CH deformation (1268), C-N stretch (1317), ring stretch υ(C=C) (1591, 1668)	722, 844, 987, 1172, 1190, 1386, 1420, 1446, 1463	Skeletal ring deformations (722); Pseudo symmetric CON stretch (844); Trigonal ring breathing (987); υ_s_(S=O) (1172); υ_s_(S=O) (1190); CH_3_ symmetric deformation (1386); υ_s_(CO_2_^-^) (1420); CH_3_, OCH_2_ deformations (1446); CH3 deformation (1463)	[29,30]
Chlorpyrifos	628, 1240, 1278, 1567	P=S (628); Cl-ring, δ(C–H), υ(P-O), Cl–H (1240); Cl-ring, δ(C-H), *ν*_as_(C=C) (1278); Ring stretching mode, υ(C=C) (1567)	673, 1390, 1404, 1437, 1450,1545	υ(C–Cl), P=S (673); Cl-ring, ν(C–N), δ(C–H) (1390–1404); Cl-ring, ν(C=C), Ring vibrations (1437); C–H def., Cl–ring, υ(C=C) (1450); Ring stretching vibrations (1545)	[31,32,33,34,35,36,37,38]
Thiamethoxam	611, 650, 687, 959, 1411	υ(C-S-Cl), υ(C-S) (611); υ(C-S), w(C-N-C-H) (650); w(C-H), w(C-N), υ(C-S) (687); σ(C-H) (959); w(Methylene) (1411)	594, 628, 667, 743, 769, 990, 1190, 1244, 1424–32, 1489, 1518–28,	w(C-N), w(H-N-C), w(C-N-C-S) (594); υ(C-S), w(C-N-C-H), w(C-N) (628); w(O-C-N-C), w(C-N-C-H), w(C-N), υ(C-S) (667); w(C-N-N-O), w(N-C-N-N) (743); w(C-N-N-O) (769); υ(O-N-O), υ(N-N), w(H-C-O-C), υ(C-O-C) (990); σ(H-C-O), σ(H-C-N), ρ(CH_3_) (1190); w(H-C-N-C) (1244); σ(H-C-O), σ(H-C-N) (1424–32); σ(H-C-H), w(H-C-N-C) (1489); υ(C=N), σ(H-C-H), σ(H-C-O), w(H-C-N-C) (1518–28)	[39,40,41,42]
Acetamiprid	621, 1416, 1491,1583	υ(C-Cl) (621); υ_s_(CO_2_) (1416); Ring structure (1491); ν(C=C) within phenyl rings (1583)	635, 775, 810–20, 1099, 1289, 1342, 1426, 1561	Ring structure and rocking (635); γ=CH within phenyl rings (775); γ=CH within phenyl rings (810); Ring rocking (820); Ring mode (P—C) (1099); w(CH) (1289); R-SO_2_-OH (1342); δCH_2_ within phenyl rings (1426); ν(C=C) (1561)	[30,42,43,44,45,46,47,48,49,50,51]
Cypermethrin	1280, 1597, 1614	υ(C = N) (1280); υ(C=C), ν(O=C-O) (1597); Ring vibrations, υ(C=O) (1614)	644, 658, 918, 1388, 1416, 1581	R-C = C-H (644); υ_s_(C-Cl) (658); n(C–C) (918); Ring stretch (1388); Ring stretch (1416); υ(C=C), ν(O=C-O) (1581)	[42,50,52,53,54,55]
Ametoctradin	614, 664, 1308, 1340, 1504	Skeletal ring deformations (614, 664); aromatic C-N stretch (1308, 1340); ring stretch δ(C-C) (1504)	1260, 1433–43, 1470, 1502, 1554	Ring stretch (1260); Ring stretch (1433–43); CH_3_, OCH_2_ deformations (1470); Ring stretch (1502); υ_as_(NO_2_) (1554)	[30]
Imidacloprid	628, 746, 811, 955, 994, 1109, 1278, 1479, 1580,	δ(C-N), δ(C-C) (628); δ(N-O), δ(C-N), υ(C-C), ρ(C-H) (746); δ(C-N), υ(C-C), υ(C-Cl), ρ(C-H) (811); υ(C-C) (955); δ(C-N), δ(C-N) (994), δ(C-H), υ(C-Cl) (1109); υ(N-O), τ(C-H), w(C-H), δ(C-H) (1278); δ(C-H) (1479), υ(C-C) (1580)	769, 825, 880, 975, 1050, 1096, 1141, 1193, 1234–46, 1293, 1368, 1443, 1468, 1548, 1563	δ(N-O), δ(C-N), υ(C-C), ρ(C-H) (769); δ(C-N), υ(C-C), υ(C-Cl), ρ(C-H) (825); Ring sym. def. (880); Trigonal ring breathing (975); υ(C-C) (1050); υ(C–N) (1096); δ(C-C), w(C-H) (1141), τ(C-H) (1193); τ(C-C), w(C-H), υ(C-N), υ(N-N) (1234–46); w(C-H), δ(C-H), υ(N-O) (1293); υ(C-C), υ(C-N), w(C-H) (1368); υ(C-C), υ(C-N), δ(C-H) (1443); υ(C=C) (1468); υ(C-C) (1548); υ_as_(NO_2_) (1563)	[11,30,39,42,56,57]
Etoxazole	592, 605, 692, 752, 811, 1662	w(CH) (592); CH (605); Symmetric skeletal stretch, C=S stretch (692); Symmetric skeletal stretch (752); Ring breathing (811); υ_s_(C=N) (1662)	563, 635, 706, 736, 801, 828, 895, 971, 1017, 1044, 1053, 1100–09, 1204, 1278, 1604, 1620	υ(CCl) (563); HH (635); Ring vibration (706); Ring vibration (736); Ring breathing (801); Ring vibration (828); Symmetric CNC stretch (895); υ(P—N) (971); υ(C–C) (1017); S=O stretch (1044); Ring vibration (1053); Trigonal ring breathing (1100–09); C_6_H_5_–C vibration (1204); Ring stretch (1278); CDC stretch (1604); Three or more coupled C=C stretches (1620)	[30,42]
Dimethomorph	627, 679, 699, 756, 1087, 1134, 1584, 1607	R-CHR-NO_2_ (627); υ(S-N) (679); υ(S-N) (699); Phenyl ring vibrations (700–1000); υ(C-O) (1000–1260); υ(C–C) vibrations (1585–1600)	841, 867, 1014, 1113, 1180, 1195, 1256	ρ(CH_3_) (841); Ring breathing (867); υ(C-O) (1000–1260)	[42,58]
Chlormequat Chloride	775, 1447, 1460, 1475	υ(C-Cl) vibration (775); CH_2_ oscillating vibration (1447); CH_3_ deformation (1460); CH_3_, OCH_2_ deformations (1475)	1263, 1295, 1342, 1390, 1414	Ring breathing (1263); In-plane CH deformation (1295); υ_s_(CO_2_^−^) (1342); Ring stretch (1390); υ_s_(CO_2_^−^) (1414)	[30,59]
Pyrimethanil	992, 1298, 1599	Breathing mode aromatic ring (992); Antisymmetric stretching (1298); CH ring antisymmetric stretching (1600)	562, 1252, 1560	Antisymmetric stretching (562); Pyrimidine in-plane deformation (1252); Antisymmetric stretching (1560)	[60,61]
Famoxadone	642, 743, 1024, 1076, 1603	Skeletal ring deformations (642, 743); ortho-substituted aromatic ring stretch (1024, 1076); mono-substituted aromatic ring stretch (1603)	1021, 1077, 1190, 1257, 1445	Ring vibration (1021); υ(S=O) stretch (1077); υ_s_(SO_2_) (1190); Amide III band (1257); Ring stretch (1445)	[30]
Tebuconazole	634, 805, 823, 1089, 1231, 1593	δ_as_(phenyl moiety) (634); δ_w_(C-H phenyl moiety), δ_σ_(C-C), γ(C-N) (805); δ_w_(C-H phenyl moiety), υ(C-C), υ(C-C) (823); υ(C-C), δ(OH), δ_ρ_(CH_3_) (1089); δ(C-H) (1231); υ_as_(C-C phenyl moiety), δ_τ_(CH_2_) (1593)	585, 684, 847, 877, 1008, 1131, 1175, 1214, 1272, 1293, 1319, 1350, 1361, 1374, 1433, 1468	υ(CH) (585); δ(phenyl moiety); δ_ρ_(CH_2_), υ(C-Cl), γ(triazole moiety) (684); γ(phenyl moiety), δ_ρ_(C-C) (847); δ_τ_(phenyl moiety) (877); γ(phenyl moiety) (1008); υ(C-C), δ_ρ_(CH_2_), δ_ρ_(CH_3_), δ(C-H), υ(C-N) (1131); υ(C-N-C); δ(C-H) (1175); δ_τ_(CH_2_), δ(OH), δ(C-H) (1214), δ_τ_(CH_2_), δ(OH) (1272–1293–1319); υ_as_(C-C-C), δ(C-H) (1350–1361–1374); υ_as_(N-C-N-C), δ_τ_(CH_2_), δ_w_(CH_3_) (1433); υ_as_(C-C-C-C), δ(C-H), δ_τ_(CH_2_) (1468)	[42,62]
Boscalid	740, 758, 913, 1041, 1050, 1086, 1298, 1600	C-Cl vibration, υ(C–H) vibration (600–1000), Ring vibration (1041); υ(S=O) (1050); υ_s_(SO_2_) (1086); CC bridge bond stretch (1298); δ(N–H) vibrations (1605)	628, 647–54, 1063, 1167, 1185, 1269	υ(C-Cl), C-Cl vibration, υ(C–H) vibration (600–1000); υ(S=O) (1063); υ_s_(SO_2_) (1167); υ_s_(SO_2_) (1185); In-plane CH deformation (1269)	[30,63,64]

## Data Availability

The baseline corrected, normalized, and averaged data (n ≥ 20) for all pesticides from both instruments (785 nm and 532 nm Raman Instruments) were provided publicly on the website given below. The algorithm used for the machine learning study was also publicly available on the same website link: https://github.com/meralyucekurt/Raman-Fingerprints-of-Pesticides (accessed on 25 February 2025).

## Supplementary Materials

The following supporting information can be downloaded at: https://www.mdpi.com/article/10.3390/bios15030168/s1, Figure S1: Baseline Correction Methods, Figure S2: Peak Selection for Metalayxl.
