# Supplementary material for: Comprehensive Raman Fingerprinting and Machine Learning-Based Classification of 14 Pesticides Using a 785 nm Custom Raman Instrument
Source: Biosensors (Basel). 2025 Mar 5;15(3):168. doi: 10.3390/bios15030168 (PMC11940532; doi:10.3390/bios15030168)
Supplement: Supplementary file 1 [file biosensors-15-00168-s001.zip › biosensors-3420792-supplementary.pdf]

COMPREHENSIVE RAMAN FINGERPRINTING AND MACHINE LEARNING-BASED CLASSIFICATION OF 14 PESTICIDES USING A 785 NM CUSTOM RAMAN INSTRUMENT

Meral Yüce[1,5,*], Nazlı Öncer[1], Ceren Duru Çınar[2], Beyza Nur Günaydın[1,3,ł], Zeynep İdil Akçora[4,ł], Hasan Kurt[5,*]

[1] SUNUM Nanotechnology Research and Application Centre, Sabanci University, Istanbul, 34956, TR
[2] Department of Computer Science & Engineering, Sabanci University, Istanbul, 34956, TR
[3] Department of Materials Science and Nanoengineering, Sabanci University, 34956, TR
[4] Department of Molecular Biology, Genetics and Bioengineering, Sabanci University, 34956, TR
[5] Department of Bioengineering, Royal School of Mines, Imperial College London, London, SW7 2AZ, UK
ł Equal contributions

[*]**Corresponding authors:** meralyuce@sabanciuniv.edu, m.yuce@imperial.ac.uk, and h.kurt@imperial.ac.uk

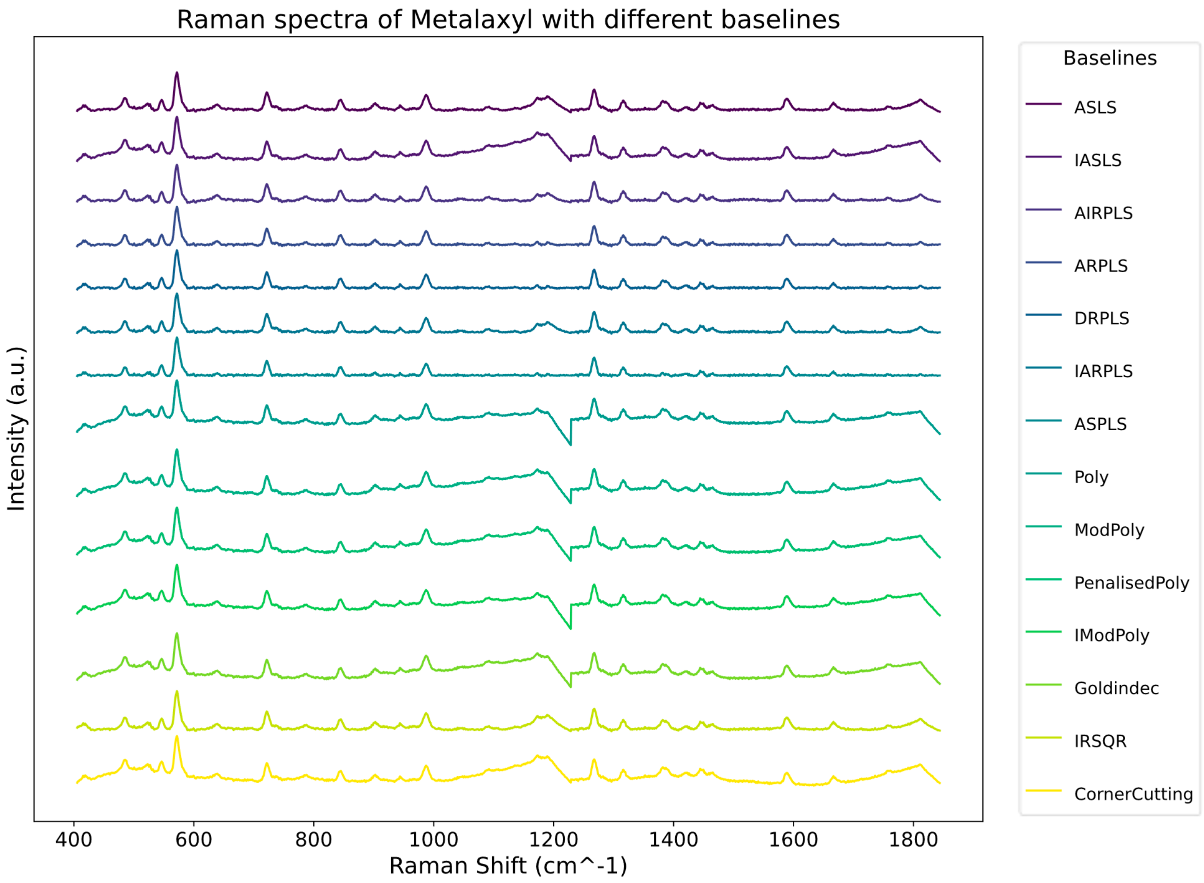

Figure S1. Baseline-corrected Metalaxyl ort spectra obtained using different correction methods (Asymmetric Least Squares Smoothing, ASLS; Iterative Asymmetric Least Squares, IASLS; Adaptive Iteratively Reweighted Penalized Least Squares, AIRPLS; Asymmetrically Reweighted Penalized Least Squares, ARPLS; Double Reweighted Penalized Least Squares, DRPLS; Iterative Asymmetric Reweighted Penalized Least Squares, IARPLS; Asymmetric Smooth Penalized Least Squares, ASPLS; Polynomial Baseline Correction, Poly; Modified Polynomial Baseline Correction, ModPoly; Penalized Polynomial Baseline Correction, Penalized; Iterative Modified Polynomial Baseline Correction, IModPoly; Goldindec Baseline Correction, Goldindec; Iterative Robust Smoothing Quadratic Regression, IRSQR; Corner Cutting Baseline Correction, CornerCutting).

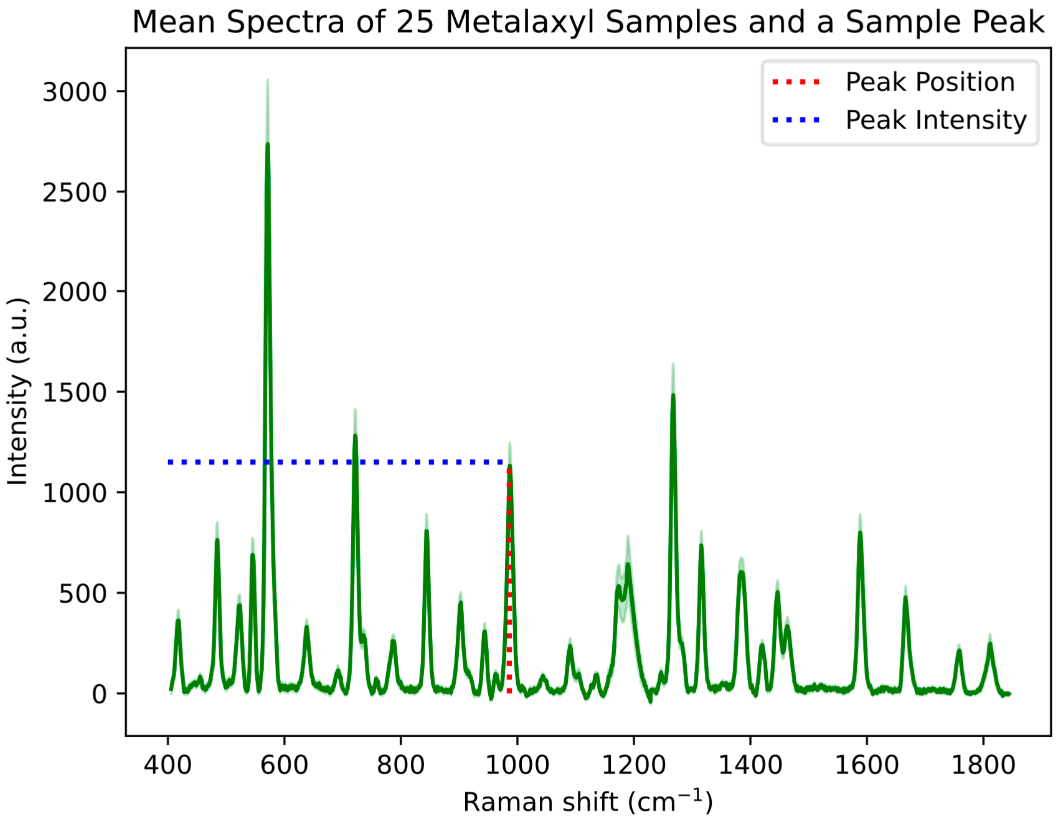

Figure S2. Selection of the three highest peaks in each 200 cm-1 Raman shift range of the Metalaxyl pesti
